# Localization of premature ventricular contraction foci in normal individuals based on multichannel electrocardiogram signals processing

**DOI:** 10.1186/2193-1801-2-486

**Published:** 2013-09-25

**Authors:** Sima Soheilykhah, Ali Sheikhani, Alireza Ghorbani Sharif, Mohammad M Daevaeiha

**Affiliations:** Department of Biomedical Engineering, Islamic Azad University, Science and Research branch, Tehran, Iran; Assistant Professor of Department of Biomedical Engineering, Islamic Azad University, Science and Research branch, Tehran, Iran; Cardiologist and Electrophysiologist of Tehran Arrhythmia Center, Tehran, Iran; Non-invasive cardiac electrophysiology research laboratory, Day general hospital, Tehran, Iran

**Keywords:** Electrocardiogram, Premature ventricular beats, PVC foci (focuses)

## Abstract

A premature ventricular contraction (PVC) is relatively a common event where the heartbeat is initiated by the other pathway rather than by the Sinoatrial node, the normal heartbeat initiator. Determining PVC foci is important for ablation procedure and it can help in pre-procedural planning and potentially may improve ablation outcome.

In this study, 12-lead Electrocardiogram (ECG) of 87 patients without structural cardiac diseases, who had experienced PVC, were obtained. Initially, PVC foci were labeled based on Electrophysiology study (EPS) reports. PVC beats were detected by wavelet method and their foci were classified using Mahalanobis distance and One-way ANOVA. Using morphological, frequency and spectrogram features, these foci in the heart were classified into five groups: Left Ventricular Outflow Tract (LVOT), Right Ventricular Outflow Tract (RVOT) septum, basal Right Ventricular (RV), RVOT free-wall, and Aortic Cusp (AC).

The results showed that 88.4% of patients are classified correctly.

**Electronic supplementary material:**

The online version of this article (doi:10.1186/2193-1801-2-486) contains supplementary material, which is available to authorized users.

## Introduction

Premature ventricular contraction (PVC) is the most common cardiac arrhythmia in patients with or without any kind of diagnosed cardiac diseases (Chiu et al. 2005). PVC is an extra heart beat originates from the ventricles and comes before the normal heart beat. Although in general, this arrhythmia may occur in a healthy person, but it is mostly associated with elderly patients (males: 60-80%) (Nathani et al. 2007) and patients suffering cardiac diseases such as hypertension, myocardial infarction, and so on (Chikh et al. 2010).

Today, the electrocardiogram (ECG) still remains the simplest and cost effective non-invasive diagnostic method for determining arrhythmias. Physicians interpret the morphology of the ECG waveform and decide whether the heartbeat belongs to the normal sinus rhythm or to the class of cardiac arrhythmias. Thus it is useful to localize the site of origin of PVCs before ablation procedure. This helps not only in pre-procedural planning, but also can potentially improve ablation outcomes (Lin et al. 2008).

PVCs may originate from various foci. If PVC focus is in right ventricle, it would appear as Left Bundle Branch Block (LBBB) and if it is in left ventricle, it would appear as Right Bundle Branch Block (RBBB) because in this state left ventricle would depolarize earlier. In general there are three common regions are defined for PVC foci: Right Ventricular Outflow Tract (RVOT), Left Ventricular Outflow Tract (LVOT) (Betensky et al. 2011) and Aortic Cusp (AC). Many researches for determining various divisions of idiopathic VT or PVC foci have been developed so far, including idiopathic ventricular tachycardia (IVT) consist of RVOT VT/PVC, Idiopathic Left Ventricular Tachycardia (ILVT), Idiopathic Propranolol sensitive VT (IPVT), LVOT VT/PVC (Nathani et al. 2007) and AC (Lin et al. 2008). It has been reported that 60-80% of the idiopathic tachycardia in normal hearts arise from the RVOT and 10% of them arise from LVOT (Lerman et al. 1997). RVOT VT/PVC is more common in females at age 30 to 50 years old (Nakagawa et al. 2002) with wide QRS complex and LBBB pattern in inferior axis (Shin et al. 2007), whereas LVOT VT/PVC usually shows RBBB morphology in lead V1 with wide monophasic R-wave in precordial leads. Morphologic explanations of ECG characteristics are useful for differentiating of VT/PVC arising from the AC region. VT/PVC originated from the left coronary cusp produces multiphasic QRS morphology with an M or W configuration in lead V1 with a precordial transition no later than V2. A left bundle pattern with a wide small R wave in lead V2 and a precordial transition usually at V3 is revealed in PVCs with a right coronary cusp origin (Lin et al. 2008).

Kamakura et al. (Kamakura et al. 1998) proposed the method to estimate the origin of VT/PVC from the RVOT and LVOT by using indexes obtained from 12-lead ECG. They classified PVC/VT from the RVOT into 8 subdivisions by using 3-dimensional anatomic relation: anterior-posterior, right-left, and superior-inferior. The features they used for estimating the origin of PVC/VT consisted of morphology, amplitude, duration and polarity of QRS complex. To distinguish LVOT from RVOT region, they showed that R/S amplitude ratio in lead V_3_ is a helpful index. If the ratio of R/S amplitude in V_3_ is equal or higher than 1, the PVC/VT stems from LVOT zone, otherwise arises from RVOT.

The aim of this study is classifying PVCs and finding their foci using multichannel ECG signals processing and various features such as morphological, frequency and spectrogram features.

## Materials and methods

### Data collection

12 lead ECGs of the 90 patients without structural cardiac disease, in whom PVC or VT had been ablated successfully, were used. The data was collected in Tehran Arrhythmia Clinic. The PVC foci were confirmed during the ablation procedure and attached as a label of data. Based on EPS reports, foci were divided into six subgroups including: LVOT (anterior & posterior), RVOT septum, basal RV, RVOT free-wall, Aortic cusp (Left Coronary cusp, Right Coronary cusp & Non-Coronary cusp) and LV body.

According to the EPS reports, 8 out of all PVCs were originated from the LVOT. Of the 52 PVCs originating from the RVOT, 15 were grouped as of the free-wall origin, 37 of the septum zone. 18 of PVCs had originated from the aortic cusp. 3 of PVCs were arisen from the LV body. The origins of remained PVCs were identified as the basal RV. Due to the lack of patients whose PVCs originating in LV body (there were only 3 patients), this group was omitted and study was done with remaining 87 patients.

**Figure 1:**
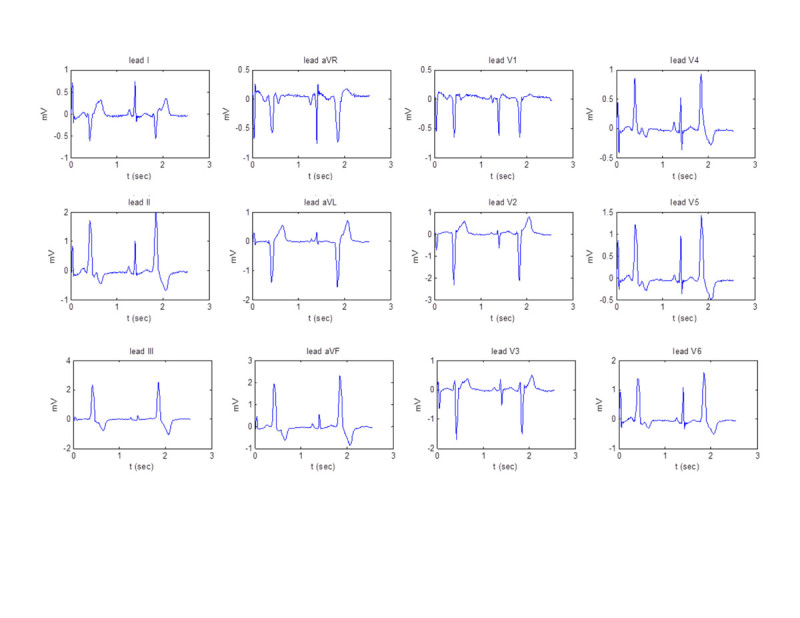
**A sample of 12-lead ECG signal of a patient with PVC originated from RVOT septum.**

Figure 1 shows a sample of 12-lead ECG of patients with PVC originating from RVOT septum.

### PVC detection

First of all, PVCs are recognized and distinguished from the normal beats. Because of their greatness in height, depth and length, PVCs could easily be detected. Since 12 leads ECG signals were recorded simultaneously, one lead was used to detect duration of the PVC beat and finally the PVC beats were extracted in all leads. Because PVC was more obvious than normal one in lead III, this lead was used for detecting PVC heartbeats.

In order to attenuate high frequency components, the ECG signal was filtered using a low pass equiripple finite-duration impulse response (FIR) filter with cut-off frequency at 25 Hz implemented with MATLAB 7.8.0 (R2009a) (The Mathworks, Inc.). In this way, considering lower frequency content of PVCs, beats would become more obvious than normal one and would be detected easier.

The standard parameters of the ECG waveform can be determined with high accuracy using wavelet transforms (Sumathi & Sanavullah 2009; Bensegueni & Bennia 2012). The wavelet transform (WT) provides a representation of the signal in time-scale domain, allowing representation of the temporal features of a signal at different resolutions. Detection of these main points is based on maximum absolute values and zero crossing of WTs at specific scales (Chang et al. 2013).

The Haar wavelet was used in our study at scale 2^1^ for PVC detection (Sumathi & Sanavullah 2009). R peak in PVC beat is detected by marking the zero crossing of the WT between positive maximum-negative minimum pair (Martínez et al. 2004). Ascendant edge of the wave is relevant to negative minimum and decreasing edge of wave is relevant to positive maximum.

First we chose a peak greater than the threshold (0.1) and then absolute of WT signal was calculated. Then we determined the nearest peaks before and after the chosen peak and finally selected the greater one. The first minimum before the initial peak and the first minimum after the second peak were specified as onset and offset of PVC beat. Because of simultaneous recording of ECG leads, duration of onset and offset of PVC beat was assigned to other leads and PVCs were determined in all leads.

### Features extraction

In this study we extracted the morphological, frequency and spectrogram features after PVCs detection to classify their five foci. Since a physician classifies arrhythmia with the information of rhythm and morphology, an input vector can consist of features that illustrate the rhythm and morphology properly (Song et al. 2005).

**Figure 2:**
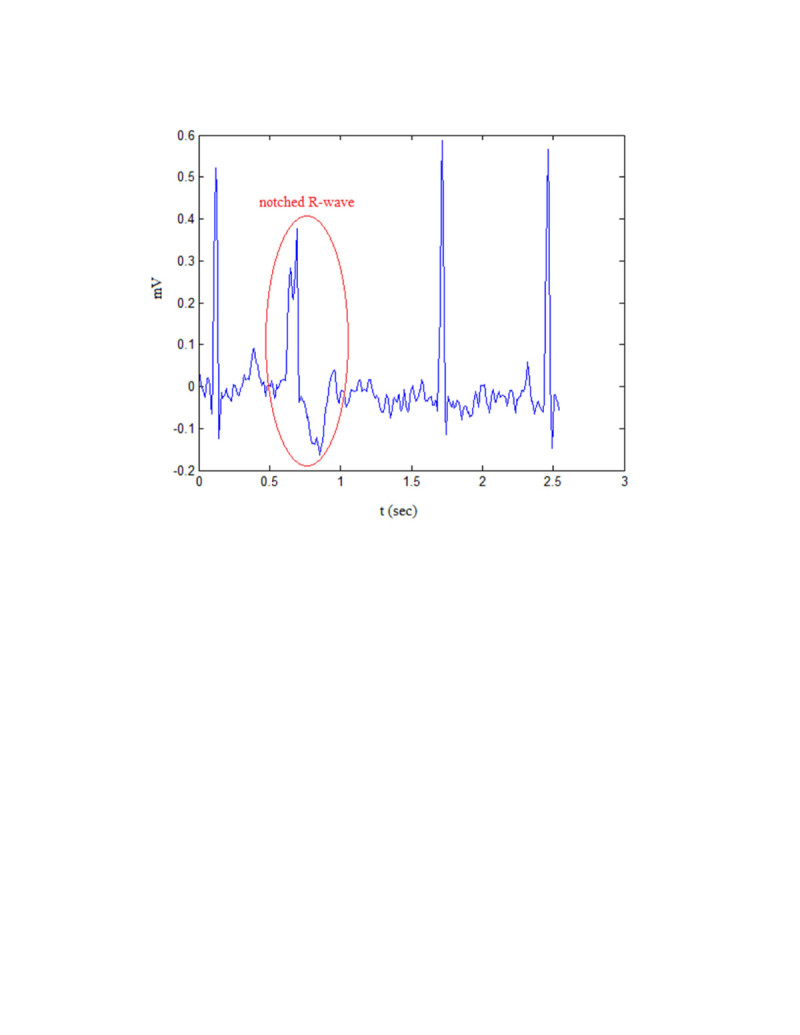
**A sample of notched QRS complex.**

One of the morphological features is polarity of each-lead ECG signal. The positive, biphasic or negative polarity was considered with 1, 0, or −1 respectively. Existence or absence of notching (Yamashina et al. 2011) in signal was shown with 1 or 0. Notching in QRS complex is determined as a tri-phasic R or Q wave with an interval greater than 40 m-sec between the first and second peak of the QRS complex. Existence of notching is considered when notching is observed in more than three of the six limb leads. As it was obtained, notching was observed more often in the PVCs arising from the free-wall rather than in the PVCs originating from the septal region (Tada et al. 2007). Figure 2 shows a sample of notched QRS complex.

Spectrum content of ECG signal in various leads is another feature that is used in classification of PVCs in this study. Frequency band of P and T waves in lead II, the most applicable lead, is approximately 0.5 to 10 Hz and QRS complex has frequencies ranging approximately from 3 to 40 Hz. Heart rate dependencies of waves in ECG must be taken into consideration. Heart rate changes will cause changes in waveforms and frequency contents. So some frequency components of ECG signal can be defined as classifying features and Fast Fourier Transform (FFT) was used for this purpose. Fourier transform of a signal and its inverse are calculated by following equations (Subha et al. 2010).

1 $$X\left(f\right)=\int_{-\infty }^{\infty }x\left(t\right)e^{-j2\pi \mathrm{ft}}\mathrm{dt}$$

2 $$x\left(t\right)=\int_{-\infty }^{\infty }X\left(f\right)e^{j2\pi \mathrm{ft}}\mathrm{df}$$

In these equations, *t* represents time and *f* denotes frequency, *x*(*t*) is original signal in time domain and *X*(*f*) represents its Fourier transform in frequency domain.

The Short-time Fourier transform (STFT) is obtained from the Fourier transform by multiplying the time signal *x*(*t*) by a window function (Tokmakei & Erdogan 2009; Hardalac et al. 2007).

3 $$\mathrm{STFT}\left(\omega ,\tau \right)=\int_{-\infty }^{+\infty }x\left(t\right)\Psi^{{}^{*}}\left(t-\tau \right)e^{-\mathrm{j\omega \tau}}\mathrm{dt}$$

Spectrogram, magnitude of STFT, shows frequency characteristics of signals in the time domain. The average of spectrogram greater than the 70 percent (Sheikkani et al. 2012) was computed for all leads and used as a feature for separating the groups.

The other spectral features are as follows: Maximum amplitude of spectrum signals, variance of signals’ spectrum, average of FFT of signal, power spectrum and power spectrum bandwidth of signals, the average of amplitudes of spectrum signals greater than the 70 percent and the average of Haar wavelet coefficients at level 1 and 2.

### Statistical analysis and classification

Since the assumptions of normal distribution and similarities were valid, statistical analyses of One-Way ANOVA were performed in SPSS 17.0 software to evaluate differences of features. The p< 0.05 was considered to show significant differences. Mahalanobis distance (Yeh 2009) was used for classifying the five groups by considering morphological, spectrogram and frequency features.

To determine the separation ability of pairwise groups, we used SVM classifier in MATLAB. Support Vector Machines (SVM) are supervised learning models with associated learning algorithm that analyze data and recognize patterns, used for classification. The basic SVM takes a set of input data and predicts, for each given input, which of two possible classes forms the output. It is more considerable using SVM classifier than Mahalanobis distance to discriminate two groups. We set data and their label of each group in separate matrixes, defined train and test data, then classified two groups each time and computed errors and compared them.

## Results

**Figure 3:**
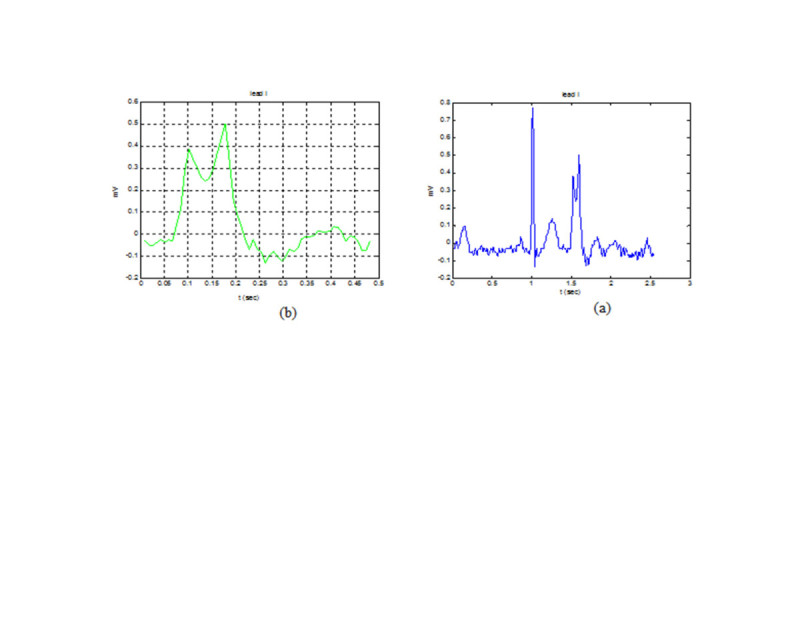
**ECG waveforms of a patient with RVOT septum PVC in lead I (a) and its PVC detected (b).**

The PVC beats were extracted from 12-lead ECG signals using the wavelet. Figure 3 shows one lead of ECG signal and extracted PVC relevant to a sample of the patients whose PVC arose from RVOT septum.

Using one-way ANOVA, the *p*-values of the 12 leads of ECG features between five groups, consist of LVOT, RVOT septum, basal RV, RVOT free-wall and Coronary cusp, are shown in Table 1. It was observed that wavelet features had no significant differences; however morphological features (QRS polarity) showed more significant differences than other features. The leads aVL, V2 and V3 showed the least significant differences, as illustrated in Table 1.

**Table 1:** **The statistical analysis (p-values), one-way ANOVA, of the 12 leads ECG features between the five groups**

Features leads	Lead I	Lead II	Lead III	Lead aVR	Lead aVL	Lead aVF	Lead V1	Lead V2	Lead V3	Lead V4	Lead V5	Lead V6
QRS polarity	0.019	0.001	0.001	0.001	0.001	0.001	0.001	0.001	0.001	0.001	0.001	0.001
Average of Haar wavelet coefficients at level 1	0.231	0.508	0.917	0.544	0.969	0.496	0.249	0.415	0.284	0.627	0.513	0.924
Average of Haar wavelet coefficients at level 2	0.67	0.559	0.588	0.580	0.096	0.101	0.795	0.582	0.556	0.725	0.135	0.564
Maximum amplitude of spectrum signals	0.001	0.001	0.007	0.001	0.172	0.001	0.001	0.33	0.009	0.002	0.001	0.001
Variance of signals’ spectrum	0.002	0.001	0.010	0.001	0.254	0.001	0.001	0.037	0.036	0.001	0.001	0.001
Average of FFT of signal	0.002	0.001	0.001	0.001	0.099	0.001	0.001	0.064	0.430	0.001	0.001	0.001
Power spectrum of signals	0.002	0.001	0.010	0.001	0.193	0.001	0.002	0.031	0.037	0.002	0.002	0.001
Power spectrum band width of signals	0.001	0.001	0.013	0.001	0.218	0.001	0.002	0.068	0.073	0.002	0.003	0.005
Average of amplitudes of spectrum signals greater than the 70 percent	0.085	0.002	0.160	0.009	0.171	0.001	0.001	0.309	0.373	0.009	0.290	0.032
Average of spectrogram greater than the 70 percent	0.001	0.001	0.009	0.001	0.175	0.001	0.001	0.035	0.005	0.002	0.001	0.001

Using one-way ANOVA, the *p*-values of the 12 lead ECG features between five groups are shown in this table.

By considering all the features, morphological, wavelet, frequency and spectrogram, the best classification result are achieved by 88.4% (with 0.6667 and 0.9305 sensitivity and specificity, respectively) using Mahalanobis distance. The result of classification with only the frequency features was obtained 68.1%, with spectrogram features alone and with only morphological features were 55.1% and 84.1% respectively.

Error examination revealed that the most error (obtained by using SVM classification) was associated with classification of both groups RVOT septum and aortic cusps (42.86%), while the other two groups had less errors (<15%) and rather appropriate separation results.

## Discussion

This study shows a method to identify PVCs and determine their foci by ECG signals processing. This method was carried out on ECGs of 87 patients in five groups, LVOT, RVOT septum, basal RV, RVOT free-wall and aortic cusp, using wavelet, frequency, spectrogram analysis and morphological features.

The results of statistical analyses in this study demonstrated that wavelet features have no significant values while the morphological and frequency features have the most significant difference, respectively. The best classification result of 88.4% was obtained by considering all the features. Our findings also illustrate that classification of groups 2 and 5 (RVOT septum and aortic cusp region) was not precise (42.86% error). This means that it’s difficult to differentiate PVCs origins in the RVOT septum and aortic cusp region truly due to their close anatomic location. Therefore these features cannot sufficiently distinguish these two groups and it is required to use other features.

Most of researches in this regard have used some features to differentiate among foci, while in this study various features were evaluated. The features such as QRS amplitude ratio between leads aVR/aVL and II/III, time to earliest rapid deflection in precordial leads and interval to peak of R wave in lead V2 was used to predict an epicardial origin for LV-VT/PVCs by Bazan et al. (2007). Reported ECG morphologic features, including the presence or absence of a Q wave in leads II, III and aVF, were region specific to identify epicardial origin. We used more morphological features with the other features, spectrum and spectrogram, for localizing five groups.

Dixit et al. (2005) used QRS morphology, amplitude, and duration in precordial leads to categorize the origin of VT/PVCs in medial and lateral locations along basal LV region. It has been reported that using the ECG criteria was possible to localize the site of origin of clinical arrhythmia in 83% of idiopathic VT/PVC originating from basal LV, while in this study using more features, better classification was obtained (88.4%) for five groups.

Our method to the best of our knowledge is the first to employ spectral content and spectrogram values of ECGs for classification of PVC beats and determining their foci. Based on the findings, it is concluded that use of surface ECG signals, a non-invasive method, is helpful to predict PVCs and their foci and will facilitate PVC/VT localization. The proposed method can be important for ablation procedure and may help to improve ablation outcome. As a future work, it is suggested to explore more appropriate features in order to improve the result of the classification with more data set.

## Authors’ original submitted files for images

Below are the links to the authors’ original submitted files for images. Authors’ original file for figure 1 Authors’ original file for figure 2 Authors’ original file for figure 3
