# Supplementary figures and images for: Localization of premature ventricular contraction foci in normal individuals based on multichannel electrocardiogram signals processing
Source: Springerplus. 2013 Sep 25;2(1):486. doi: 10.1186/2193-1801-2-486 (PMC3790125; doi:10.1186/2193-1801-2-486)

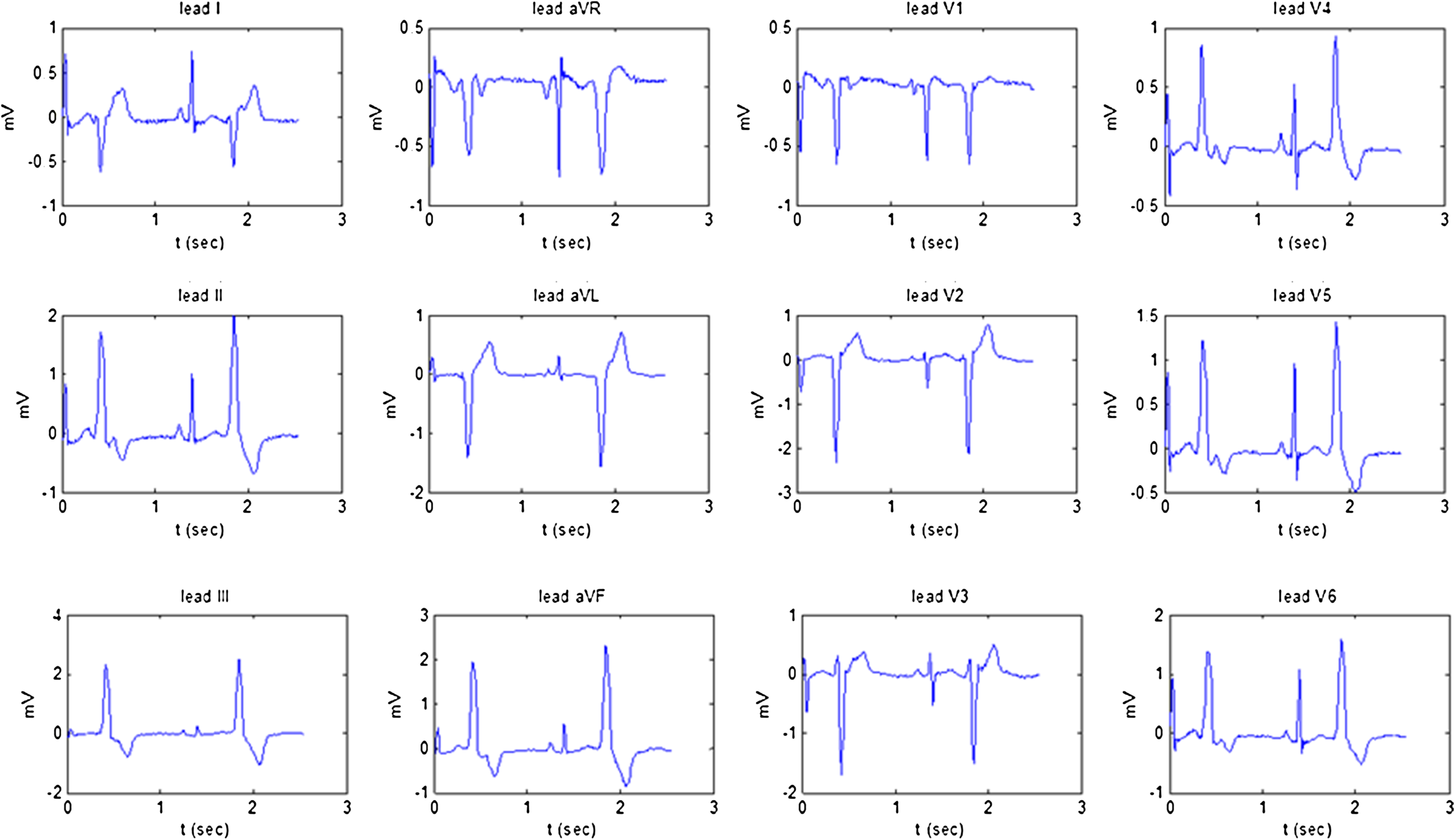

Supplement: Supplementary file 1 — Authors’ original file for figure 1 [file 40064_2013_543_MOESM1_ESM.tiff]

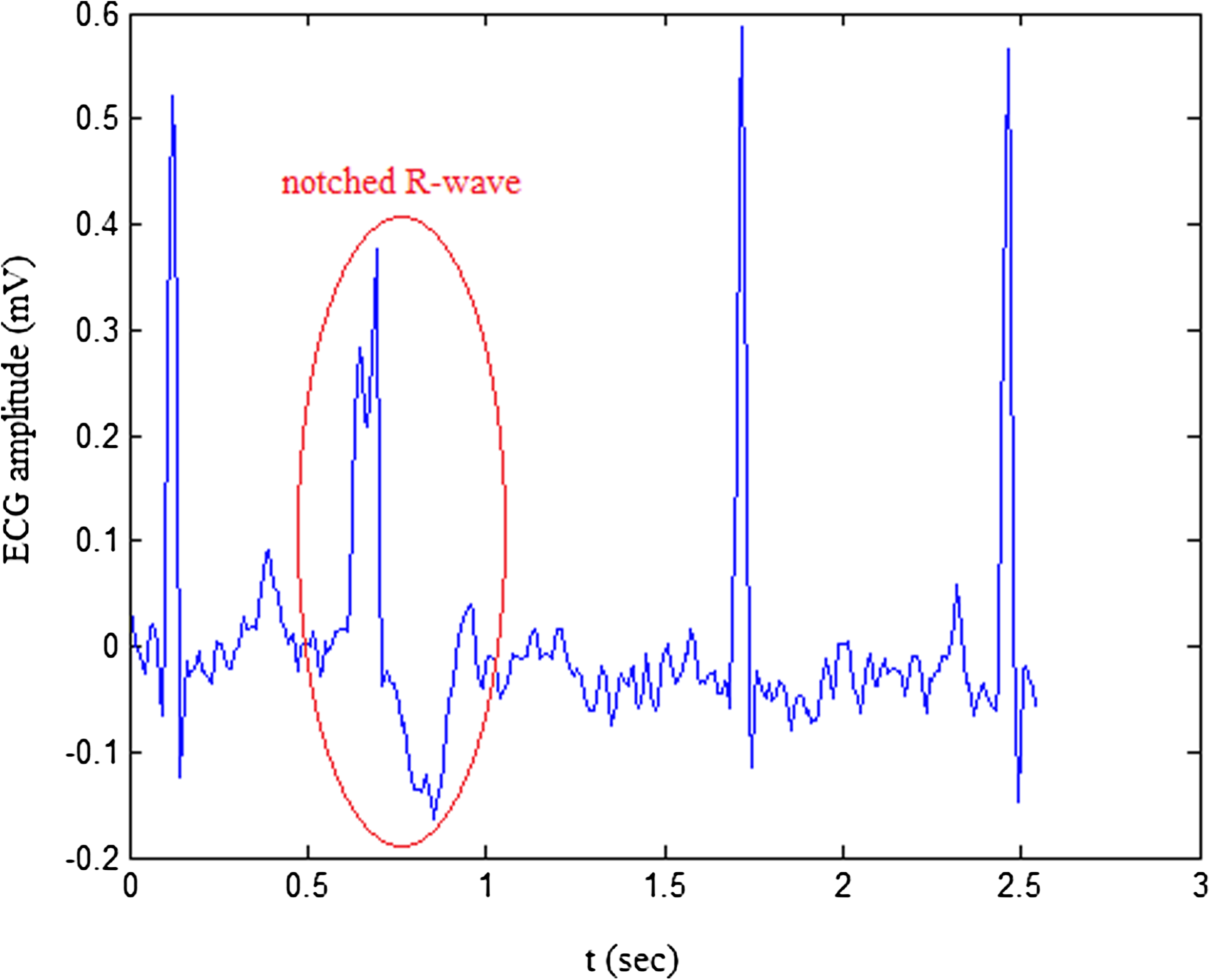

Supplement: Supplementary file 2 — Authors’ original file for figure 2 [file 40064_2013_543_MOESM2_ESM.tiff]

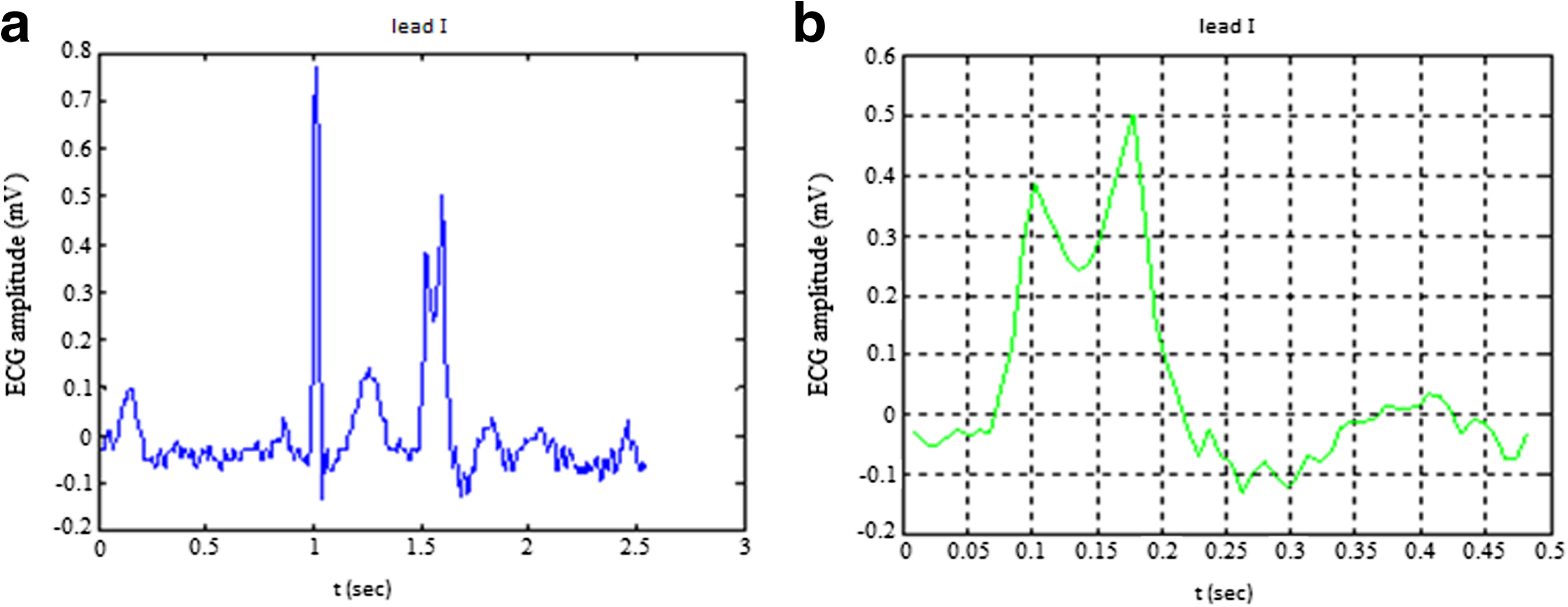

Supplement: Supplementary file 3 — Authors’ original file for figure 3 [file 40064_2013_543_MOESM3_ESM.tiff]
